# Seasonal variation and temporal relationship to the COVID-19 pandemic of NMDA receptor antibody results

**DOI:** 10.1007/s00415-023-11917-6

**Published:** 2023-09-22

**Authors:** Jonathan P. Rogers, Michael K. L. Chou, Thomas A. Pollak, Michael Eyre, Maria Krutikov, Andrew Church, Melanie S. Hart, Abid Karim, Sophia Michael, Angela Vincent, Anthony S. David, Glyn Lewis, Saiju Jacob, Michael S. Zandi

**Affiliations:** 1 Division of Psychiatry, University College London, 6th Floor, Maple House, 149 Tottenham Court Road, Bloomsbury, London, W1T 7NF UK; 2 South London and Maudsley NHS Foundation Trust, London, UK; 3 Neuroimmunology and CSF Laboratory, National Hospital for Neurology and Neurosurgery, University College London Hospitals NHS Foundation Trust, London, UK; 4 Department of Psychosis Studies, Institute of Psychiatry, Psychology and Neuroscience, King’s College London, London, UK; 5 Children’s Neurosciences, Evelina London Children’s Hospital at Guy’s and St Thomas’ NHS Foundation Trust, London, UK; 6 School of Biomedical Engineering and Imaging Sciences, King’s College London, London, UK; 7 Institute of Health Informatics, University College London, London, UK; 8 Department of Neuroinflammation, Queen Square Institute of Neurology, University College London, London, UK; 9 Institute of Immunology and Immunotherapy, University of Birmingham, Birmingham, UK; 10 Department of Neurology, University Hospitals Birmingham, Birmingham, UK; 11 Nuffield Department of Clinical Neurosciences, University of Oxford, Oxford, UK; 12 Institute of Mental Health, University College London, London, UK; 13 National Hospital for Neurology and Neurosurgery, University College London Hospitals NHS Foundation Trust, London, UK

Dear Sirs,

*N*-methyl-d-aspartate (NMDA) receptor antibody encephalitis, since its description in 2007, has emerged as one of the most common causes of encephalitis among young people in the developed world [1]. While up to a third of cases of NMDA receptor antibody encephalitis are associated with an ovarian teratoma, the aetiology of the majority of cases remains unknown [2]. However, case reports of NMDA receptor antibody encephalitis occurring in the context of infection with *Haemophilus influenzae*, human herpesvirus 6, mumps virus, *Enterovirus*, *Mycoplasma pneumoniae* and Japanese encephalitis virus have been reported [3, 4], but the most robust association is with herpes simplex encephalitis, which one study found to be followed within a year by NMDA receptor antibody encephalitis in 18% of individuals [5]. There is also evidence of an association between NMDA receptor antibodies in serum and antibodies indicative of prior influenza infection [6].

If NMDA receptor antibody encephalitis is triggered in a substantial proportion of cases by infectious agents, this has two potentially testable epidemiological implications. First, if pathogens that show a seasonal pattern in transmission are implicated, there may be seasonal variation in the incidence of NMDA receptor antibody encephalitis. One study of 126 cases from a national referral site in the Netherlands identified a peak in May and June, although this did not reach statistical significance and, in the subsequent month of July, cases were among the lowest of any month [7]. Another study of 90 NMDA receptor antibody encephalitis patients from tertiary care in China found that 60% of cases occurred in the half of the year denoted as summer or autumn [8]. In the paediatric literature, a report of 23 non-tumour-associated cases found that 78% occurred in the warmer months of April–September [9], but another study of 31 cases of NMDA receptor antibody encephalitis found no evidence of difference between summer and winter months [10]. Overall, there is a suggestion from relatively small studies that incidence might be higher in the warmer months of the year, but this is by no means conclusive and merits a larger study.

The second potential implication of an infectious trigger hypothesis is that the COVID-19 pandemic may have had an impact on incidence. There are two possible effects here, which may have acted in opposing directions. The first is that the SARS-CoV-2 virus may itself initiate NMDA receptor antibody encephalitis, driving an increase in incidence. There have been several such cases of NMDA receptor antibody encephalitis in association with COVID-19 reported in the literature [11], but given the near-ubiquity of COVID-19 at points, coincidence is a distinct possibility. The other potential effect is that the restrictions instituted by many countries could have suppressed the circulation of other infectious agents that may trigger NMDA receptor antibody encephalitis. Two studies have examined whether the COVID-19 pandemic was associated with any change in the incidence of NMDA receptor antibody encephalitis. One single-centre study that examined children with any form of autoimmune encephalitis found a fall from an average of 7.7 cases in the preceding years to 3 cases during the pandemic, representing a reduction of 61% [12]. A recent study examined 17,365 serum samples tested in a clinical laboratory for the NMDA receptor antibody, finding no change in the proportion positive during periods of pandemic-related restrictions (in contrast to LGI1 antibodies, where a reduction was observed), but this study reported no CSF results [13].

In the current study, we aimed to evaluate the variation of antibodies to the NMDA receptor in the serum and CSF across seasons of the year, and within and without the period of the COVID-19 pandemic.

We conducted a retrospective cohort study where the outcome was the number of positive NMDA receptor antibody assays. The exposure for the first part of the study was season, as measured in quarters of the year. The exposure for the second part was the period of the COVID-19 pandemic.

The study was conducted using the results from two of the largest clinical neuroimmunology laboratories in the UK: the Neuroimmunology and CSF Laboratory at University College London Hospitals NHS Foundation Trust (UCLH) and the Clinical Immunology Service at University Hospitals Birmingham (UHB). It was approved internally as a service evaluation at UCLH and an audit at UHB. Data were collected retrospectively from 1 January 2015 to 31 December 2021 with the first valid NMDA receptor antibody assay in serum and that in the CSF for a particular individual within the observation period considered eligible. The sample size was ascertained pragmatically by including all available samples.

NMDA receptor antibodies had been tested using indirect immunofluorescence with the commercially available Euroimmun fixed-cell assay. Antibody results were dichotomised as positive (weakly positive, positive and strongly positive) and negative. Equivocal results were excluded. Results for serum and CSF antibody assays were analysed separately. As it is not clear what the latency from infection to encephalitis might be, a broad pandemic window of all of the calendar years of 2020 and 2021 was used.

Percentages of positive and negative test results were calculated during each time period. In estimating seasonality, to predict the number of positive assay results as the dependent variable, a negative binomial regression model was used, as the equidispersion assumption did not hold. In model 1, the independent variable was quarter of the year across the 7 years of data, making 28 data points. Quarter was used as a categorical variable with Q1 being January–March, Q2 April–June, Q3 July–September, and Q4 October–December. Q1 was used as the reference category. In model 2, to account for longitudinal trends over time, sample year was added as a continuous covariate. In model 3, to account for changing practices in ordering tests, the number of valid tests ordered in the quarter (positive and negative) was added as a further covariate. The fully adjusted model (model 3) was considered the primary analysis. The likelihood ratio test was used to generate *p*-values for seasonal variation in each of the models with and without the quarter of the year.

To analyse whether there was any impact of the pandemic, the proportion of test results that were positive during 2020 and 2021 was compared to the proportion of test results in 2018 and 2019 using a risk ratio with 95% confidence intervals and the chi-squared test. Given that we effectively only had two exposure periods, a more sophisticated model such as that used for seasonality was not possible.

Analysis was conducted in Stata/MP 17.0 and statistical significance was set to 0.05.

A total of 12,464 laboratory results meeting the inclusion criteria were extracted, 4854 from UCLH and 7610 from UHB. 10,296 results were from serum and 2168 were from CSF. Linkage between serum and CSF samples was available only for the UCLH samples, showing that among 129 positive results in serum, only 17 (13.2%) also had CSF testing, of which 15 were positive and 2 negative.

The results for the analysis of seasonality using quarters of the year are shown in Table 1. The coefficients for all covariates are shown in Supplementary Tables 1 and 2.

**Table 1:** Total numbers of test results and negative binomial regression for seasonality

	Quarter	Total results, *n* (%)	Positive results, *n* (%)	Negative results, *n* (%)	Model 1^a^		Model 2^b^		Model 3^c^	
					IRR (95% CI)	*p*	IRR (95% CI)	*p*	IRR (95% CI)	*p*
Serum	Q1 (ref)	2466 (24.0)	76 (23.4)	2390 (24.0)	–	–	–	–	–	–
	Q2	2499 (24.3)	74 (22.8)	2425 (24.3)	0.97 (0.67–1.41)	0.89	0.97 (0.69–1.36)	0.87)	0.94 (0.68–1.30)	0.70
	Q3	2524 (24.5)	91 (28.0)	2433 (24.4)	1.20 (0.84–1.71)	0.33	1.20 (0.87–1.65)	0.27	1.16 (0.86–1.58)	0.33
	Q4	2807 (27.3)	84 (25.8)	2723 (27.3)	1.11 (0.77–1.59)	0.59	1.11 (1.02–1.14)	0.54	0.97 (0.69–1.35)	0.84
	Overall seasonal effect	–	–	–	–	0.67	–	0.56	–	0.52
CSF	Q1 (ref)	483 (22.3)	16 (18.4)	467	–	–	–	–	–	–
	Q2	517 (23.8)	21 (24.1)	496 (22.4)	1.31 (0.60–2.89)	0.50	1.29 (0.65–2.56)	0.47	1.18 (0.61–2.29)	0.63
	Q3	552 (25.5)	31 (35.6)	521 (25.0)	1.94 (0.92–4.09)	0.08	1.94 (1.02–3.66)	0.04	1.64 (0.87–3.09)	0.13
	Q4	616 (28.4)	19 (21.8)	597 (28.7)	1.19 (0.53–2.64)	0.67	1.17 (0.58–2.35)	0.66	0.85 (0.40–1.85)	0.69
	Overall seasonal effect	–	–	–	–	0.35	–	0.20	–	0.17

Percentages indicate the proportion of assay results across the years from a particular quarter

*IRR* incidence rate ratio

^a^Negative binomial regression with number of positive results as dependent variable and quarter of the year as independent variable

^b^Model 1 with sample year added as a covariate

^c^Model 2 with total number of tests ordered added as a covariate

The evolution in the number of tests requested and the proportion positive is shown in Fig. 1. The raw numbers of positive and negative results by year are shown in Supplementary Table 3. Among serum samples, 105/3772 (2.8%) tests were positive in 2020–2021, compared to 96/3233 (3.0%) in 2018–2019, giving a risk ratio of 0.94 (95% CI 0.71–1.23, *p* = 0.64). Among CSF samples, 37/1133 (3.3%) tests were positive in 2020–2021, compared to 30/642 (4.7%) in 2018–2019, giving a risk ratio of 0.73 (95% CI 0.46–1.17, *p* = 0.19).

**Fig. 1:**
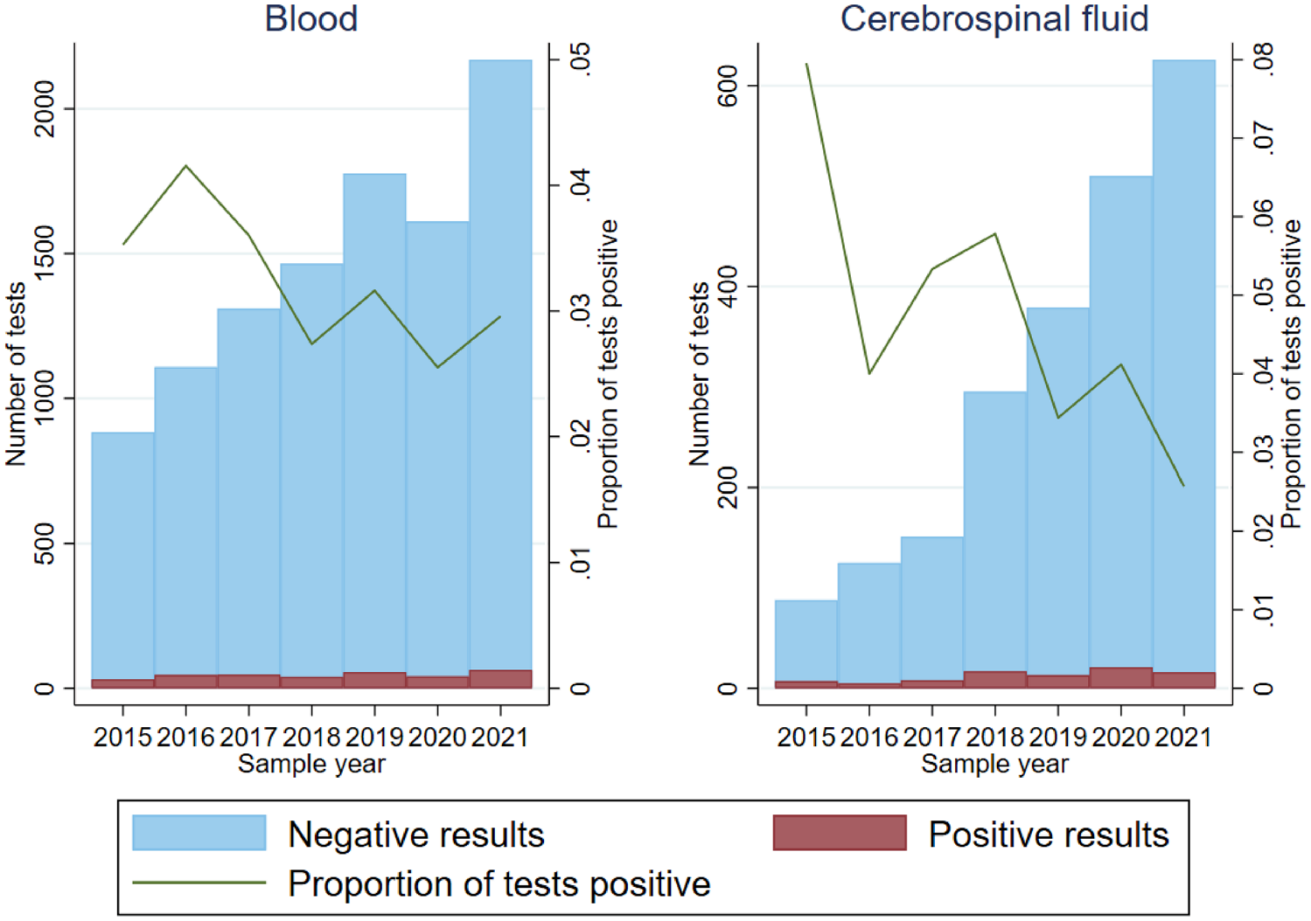
Number of tests per year and proportion of tests positive

In this retrospective study of 12,464 NMDA receptor antibody assays in serum and CSF across two large UK centres, we did not find any evidence for seasonality in the number of positive antibody results. After adjusting for sample year and the total number of tests requested, neither serum nor CSF showed a statistically significant seasonal effect. When comparing the results in the years of the pandemic to the preceding 2 years, there was no significant change in the proportion of results that were positive.

There are several limitations to this analysis. Most importantly, there are limitations in this study’s face validity in that it measured antibody results rather than actual diagnoses of NMDA receptor antibody encephalitis. The assay, particularly when measured in serum, is not entirely specific and is positive in the serum of 1.1% (95% CI 0.9–1.3%) of healthy controls [14], which may have obscured a seasonal effect in individuals with actual encephalitis, but the lower percentages despite the trend towards higher values during the pandemic years are consistent with a highly specific test. We did not have clinical and other paraclinical data available to supplement the antibody results and data were not available on further independent testing, such as immunohistochemistry. CSF IgG antibodies to the GluN1 epitope are required to support a definite diagnosis of NMDA receptor antibody encephalitis; as our results constituted only 87 positive CSF samples and not all positive serum results had confirmatory CSF testing, this study may have been insufficiently powered to detect seasonal variation.

Given that quarter of the year was a categorical variable with four levels, there were multiple tests of statistical significance in Table 1, which increases the probability of generating a positive result by chance. Therefore, the results for the overall seasonal effect should be used in preference.

There are several important limitations that may have created bias towards a null effect. One is that our identification of the first antibody result was likely to be imperfect. We used the first recorded antibody result at each laboratory in an attempt to eliminate subsequent re-tests, which would be less likely to reflect any seasonal aspect to the disease. However, it is possible that some patients had a first test prior to the start of our data collection in 2015, and some had their initial testing at another centre or seroconverted to a positive test result at a later date. The other effect that may have created bias towards a null finding would be if there were two competing effects during the COVID-19 pandemic: one increasing the incidence through cases triggered by SARS-CoV-2 and another reducing the incidence through pandemic-related restrictions inhibiting the spread of other pathogens. Third, it is more likely that it is the non-tumour-related cases of NMDA receptor antibody encephalitis that are related to infection, but our laboratory-based data did not allow us to distinguish these from the remaining cases. It is also possible that our model would not effectively have accounted for non-linear relationships with year of testing.

In terms of confounding factors, it is likely that longer term changes in patterns of ordering tests, such as an increasing number of requests—likely to represent increased clinician awareness of autoimmune encephalitides—and a diminishing proportion of positive results—possibly as a result of a lower threshold for requesting testing—as shown in Fig. 1, have been successfully dealt with in our adjusted models for seasonality, although they may remain for the assessment of a pandemic effect. However, there may be other non-disease-related seasonal effects that had an impact, such as the changes in diagnostic and therapeutic practices in hospitals related to annual changeover in doctors [15] or difficulties accessing testing services during the pandemic.

In conclusion, we may state that previous small studies finding a seasonal distribution of NMDA receptor antibody encephalitis cases may be due to chance. Our analysis, which—in contrast to many previous studies—included CSF and serum in contrast to many previous studies, did not find evidence of an overwhelming seasonal effect, although the upper bounds of the confidence intervals in Table 1 are such that we cannot exclude a smaller effect. Our results finding an absence of change in positive results during the pandemic are consistent with another similar study [13], but it extends it using the CSF results. It supports the notion that the incidence of NMDA receptor antibody encephalitis did not substantially change during this period. However, it does not rule out the possibility that SARS-CoV-2 may act as a trigger for NMDA receptor antibody encephalitis, either in a small number of cases, or with an offset in the epidemiology due to pandemic-related restrictions impacting other infectious triggers.

Our study suggests that clinicians do not currently need to have a higher index of suspicion of NMDA receptor antibody encephalitis at a particular time of year and health services do not need to differentially allocate resources based on seasonal variation.

To address this issue of seasonality with future research, studies should use NMDA receptor antibody encephalitis diagnoses, based on validated criteria and supported by antibody testing, but deep phenotyping would be required to ascertain symptom onset, as some patients may receive a diagnosis at least several weeks after first symptoms. Larger numbers of positive CSF results would be required.

### Supplementary Information

Below is the link to the electronic supplementary material.

Supplementary file1 (DOCX 19 KB)

## Data Availability

The data used in this study are not publicly available due to the information governance requirements of the respective hospitals.
