# Supplementary material for: Seasonal variation and temporal relationship to the COVID-19 pandemic of NMDA receptor antibody results
Source: J Neurol. 2023 Sep 22;270(11):5182–7. doi: 10.1007/s00415-023-11917-6 (PMC10576721; doi:10.1007/s00415-023-11917-6)
Supplement: Supplementary file 1 — Supplementary file1 (DOCX 19 KB) [file 415_2023_11917_MOESM1_ESM.docx]

# Supplementary material

## Supplementary table 1: Negative binomial regression model for seasonality for blood results with coefficients for all covariates

| Quarter | Model 1 ^a^ | | Model 2 ^b^ | | Model 3 ^c^ | |
| --- | --- | --- | --- | --- | --- | --- |
|  | IRR (95% CI) | *p* | IRR (95% CI) | *p* | IRR (95% CI) | *p* |
| Q1 (ref) | - | - | - | - | - | - |
| Q2 | 0.97 (0.67 to 1.41) | 0.89 | 0.97 (0.69 to 1.36) | 0.87) | 0.94 (0.68 to 1.30) | 0.70 |
| Q3 | 1.20 (0.84 to 1.71) | 0.33 | 1.20 (0.87 to 1.65) | 0.27 | 1.16 (0.86 to 1.58) | 0.33 |
| Q4 | 1.11 (0.77 to 1.59) | 0.59 | 1.11 (1.02 to 1.14) | 0.54 | 0.97 (0.69 to 1.35) | 0.84 |
| Sample year | - | - | 1.08 (1.02 to 1.14) | 0.01 | 0.95 (0.84 to 1.08) | 0.44 |
| Number of assays performed (in 100s) | - | - | - | - | 1.30 (1.02 to 1.65 | 0.03 |

IRR – incidence rate ratio

^a^ Negative binomial regression with number of positive results as dependent variable and quarter of the year as independent variable. ^b^ Model 1 with sample year added as a covariate. ^c^ Model 2 with total number of tests ordered added as a covariate.

## Supplementary table 2: Negative binomial regression model for seasonality for CSF results with coefficients for all covariates

| Quarter | Model 1 ^a^ | | Model 2 ^b^ | | Model 3 ^c^ | |
| --- | --- | --- | --- | --- | --- | --- |
|  | IRR (95% CI) | *p* | IRR (95% CI) | *p* | IRR (95% CI) | *p* |
| Q1 (ref) | - | - | - | - | - | - |
| Q2 | 1.31 (0.60 to 2.89) | 0.50 | 1.29 (0.65 to 2.56) | 0.47 | 1.18 (0.61 to 2.29) | 0.63 |
| Q3 | 1.94 (0.92 to 4.09) | 0.08 | 1.94 (1.02 to 3.66) | 0.04 | 1.64 (0.87 to 3.09) | 0.13 |
| Q4 | 1.19 (0.53 to 2.64) | 0.67 | 1.17 (0.58 to 2.35) | 0.66 | 0.85 (0.40 to 1.85) | 0.69 |
| Sample year | - | - | 1.21 (1.08 to 1.36) | 0.001 | 0.86 (0.56 to 1.31) | 0.47 |
| Number of assays performed (in 100s) | - | - | - | - | 4.12 (0.75 to 22.7) | 0.10 |

IRR – incidence rate ratio

^a^ Negative binomial regression with number of positive results as dependent variable and quarter of the year as independent variable. ^b^ Model 1 with sample year added as a covariate. ^c^ Model 2 with total number of tests ordered added as a covariate.

## Supplementary table 3: Numbers of positive and negative assay results by year

| Year | Serum | | | CSF | | |
| --- | --- | --- | --- | --- | --- | --- |
|  | *Total* | *Positive* | *Negative* | *Total* | *Positive* | *Negative* |
| 2015 | 879 | 31 | 848 | 88 | 7 | 81 |
| 2016 | 1107 | 46 | 1061 | 125 | 5 | 120 |
| 2017 | 1305 | 47 | 1258 | 150 | 8 | 142 |
| 2018 | 1462 | 40 | 1422 | 294 | 17 | 277 |
| 2019 | 1771 | 56 | 1715 | 378 | 13 | 365 |
| 2020 | 1608 | 41 | 1567 | 510 | 21 | 489 |
| 2021 | 2164 | 64 | 2100 | 623 | 16 | 607 |
| Total | 10296 | 325 | 9971 | 2168 | 87 | 2081 |
